# The integrated bioinformatic analysis identifies immune microenvironment-related potential biomarkers for patients with gestational diabetes mellitus

**DOI:** 10.3389/fimmu.2024.1296855

**Published:** 2024-02-21

**Authors:** Jie-ling Chen, Hui-fang Dai, Xin-chen Kan, Jie Wu, Hong-Wu Chen

**Affiliations:** ^1^ Brain Function and Disease Laboratory, Shantou University Medical College, Shantou, Guangdong, China; ^2^ Department of Physiology, Shantou University Medical College, Shantou, Guangdong, China; ^3^ Department of Neurosurgery, The First Affiliated Hospital of Shantou University Medical College, Shantou, Guangdong, China

**Keywords:** gestational diabetes mellitus, diagnostic value, immune-related hub DEGs, *PLAUR*, *SLIT2*

## Abstract

**Background:**

Gestational diabetes mellitus (GDM), a transient disease, may lead to short- or long-term adverse influences on maternal and fetal health. Therefore, its potential functions, mechanisms and related molecular biomarkers must be comprehended for the control, diagnosis and treatment of GDM.

**Methods:**

The differentially expressed genes (DEGs) were identified using GSE49524 and GSE87295 associated with GDM from the Gene Expression Omnibus database, followed by function enrichment analysis, protein-protein interactions network construction, hub DEGs mining, diagnostic value evaluation and immune infiltration analysis. Finally, hub DEGs, the strongest related to immune infiltration, were screened as immune-related biomarkers.

**Results:**

A hundred and seven DEGs were identified between patients with GDM and healthy individuals. Six hub genes with high diagnostic values, including *ALDH1A1*, *BMP4*, *EFNB2*, *MME*, *PLAUR* and *SLIT2*, were identified. Among these, two immune-related genes (*PLAUR* and *SLIT2*) with the highest absolute correlation coefficient were considered immune-related biomarkers in GDM.

**Conclusion:**

Our study provides a comprehensive analysis of GDM, which would provide a foundation for the development of diagnosis and treatment of GDM.

## Introduction

Gestational diabetes mellitus (GDM), which is the most prevalent pregnancy-related metabolic disturbance, refers to glucose intolerance that first becomes evident at some point during pregnancy (1). Approximately 2 to 10% of pregnancies in the United States and about 13.9% of pregnancies worldwide are complicated by GDM (2). The etiology of GDM is complex, owing to both genetic and environmental factors (3, 4). Several studies have revealed the critical short- and long-term adverse health consequences of GDM on both the mother and their offspring (4). Women with GDM are at risk for short- and long-term health complications, including type 2 diabetes (T2DM), cardiovascular disease (CVD) later in life and adverse cardiometabolic phenotypes in subsequent offspring (5, 6). Furthermore, GDM leads to a significant financial burden on society and healthcare resources (7, 8). Currently, the onset and progression of GDM are uncertain and complex and pathogenesis remains uncertain (9). Consequently, revealing new diagnostic and therapeutic molecular biomarkers for GDM patients for individualized and effective treatment is crucial.

The interaction between immune response and GDM has been extensively studied. The GDM’s etiopathogenesis is ambiguous, and the existing studies suggest dysregulated maternal immune systems and low -grade inflammation as critical factors in the pathophysiology of GDM (10). The maternal-fetal interface in patients with GDM demonstrates a higher proportion of cytotoxic NK cells (11) and dysregulated functions of Tregs (12, 13) and Th17 cells (14, 15) compared to normal pregnant women. Previous studies also demonstrated that immune cells and secreted cytokines might play an important role in GDM. IL-6 (16, 17), IL-1β (18), IL-38 (19) and TNF-α (18, 20) secreted by placental tissue aggravate the chronic inflammatory reaction and degree of maternal insulin resistance (IR), thus, contributing to the development of GDM. However, our understanding of the immune microenvironment in GDM is highly limited to date.

In recent years, the data generated by microarray technology have been used to study the pathophysiology of various diseases. In our study, our aim is to search for immune-related hub biomarkers with diagnostic significance for the patients with GDM through a comprehensive bioinformatic analysis, which may help develop targeted drugs for the treatment and provide a research foundation for preclinical research of GDM therapy. Firstly, an integrated bioinformatic analysis for transcriptome sequencing data of umbilical cord HUVEC cells from GDM patients was performed to identify the hub immune-related molecules or GDM biomarkers as strong evidence. The gene expression profiles of GSE49524 and GSE87295 from the Gene Expression Omnibus (GEO) database (https://www.ncbi.nlm.nih.gov/geo/) were downloaded and analyzed in this study. A hundred and seven genes (68 upregulated and 39 downregulated genes) were identified as the hub differentially expressed genes (DEGs), followed by functional enrichment analysis through Gene Ontology (GO) term, Kyoto Encyclopedia of Genes and Genomes (KEGG) pathway enrichment analysis and disease ontology (DO) enrichment analysis. Then, Six algorithms of cytoHubaa plug-in performance and Receiver operating characteristic (ROC) curve analysis, six hub DEGs with the area under the ROC curve >0.8, which have remarkable diagnostic value, after protein-protein interaction (PPI) network construction were significantly correlated with GDM. Subsequently, the relationship between these six hub genes and immune cell infiltration was analyzed. Finally, the two immune-related genes with the highest absolute correlation coefficient were screened.

## Materials and methods

### Data collection and normalization

In the present study, GDM datasets (GSE49524 and GSE87295) from the GEO database (**Table 1**) were downloaded. The GSE49524 contained three umbilical cords HUVEC sample of 3 women with GDM and three umbilical cords HUVEC sample of 3 women without GDM matching for age and Body Mass Index, and the GSE87295 contained five HUVECs samples from GDM background and five HUVECs samples from the mothers with no GDM. We pooled these two datasets and divided them into GDM group (n=8) and control group (n=8). Data preprocessing and normalization were performed through the R package “inSilicoMerging” and “combat” algorithms. The GDM datasets (GSE49524 and GSE87295) were first combined into a gene expression profile, and then the batch effect between the two datasets was removed to obtain the normalized gene expression matrix used in the subsequent analysis.

**Table 1:** Dataset characteristics of selected GEO datasets.

Number	Dataset	Platform	GDM	Normal	Total
1	GSE49524	GPL7020 (NuGO array (human) NuGO_Hs1a520180)	3	3	6
2	GSE87295	GPL10558 (Illumina HumanHT-12 V4.0 expression beadchip)	5	5	10

### Differential expression analysis

R package “Limma” was employed to screen the DEGs using |log2 Fold change (FC)| >0.5 and p < 0.05 as the cutoff criteria to investigate the differentially expressed genes (DEGs) in GDM and normal samples. The volcano and ranking plots were used to visualize the DEG distribution.

### Functional enrichment analysis

The Kyoto Encyclopedia of Genes and Genomes (KEGG) pathway enrichment analysis; Gene Ontology (GO) enrichment analysis (including biological process [BP], cellular component [CC] and molecular function [MF]); and disease ontology (DO) enrichment analysis were performed through R package “clusterProfiler” with p-value < 0.05 as the cutoff, to comprehensively obtain the functional annotation for the DEGs.

### Protein-protein interaction network analysis and hub gene identification

The STRING database (https://string-db.org) and Cytoscape software (version 3.8.2) was employed to obtain the potential PPI information and visualize the PPI network to investigate the protein-protein interaction (PPI) network of DEGs. Furthermore, the Molecular Complex Detection (MCODE) algorithm was used for the hub gene cluster analysis. Moreover, five algorithms (Degree, MNC, DMNC, EPC and MCC) of the cytoHubaa plug-in were employed to identify the hub DEGs of the PPI network in Cytoscape software. The Wilcoxon test was used to analyze the gene expression levels, and Spearman’s correlation analysis was used to depict the correlation between each of identified hub genes.

### Receiver operating characteristic curve analysis

To evaluate the accuracy of identified hub genes as the diagnostic biomarkers of patients with GDM, the ROC curve and areas under the ROC curve (AUC) were used for the evaluation of candidate biomarkers in patients with GDM through the R package “pROC”.

### Immune cell infiltration analysis

The infiltration level of 22 immune infiltrating cells in GDM and normal samples was determined using the CIBERSORT algorithm by R package “IOBR,” and the significant difference in the immune infiltration between GDM and normal samples passed the Wilcoxon test. Spearman’s correlation analysis was used to depict the correlation between each of the immune cells and between the immune cells and identified hub genes in GDM samples.

### Statistical analysis

The aforementioned bioinformatics analysis and R package were implemented using R software (v4.0.3) and Sangerbox, a comprehensive, interaction-friendly clinical bioinformatics analysis platform. Spearman’s correlation analysis was utilized to determine the correlation, and the significance of the two groups was assessed using the Wilcox test. Statistical significance was defined as p<0.05.

## Results

### Identification of differentially expressed genes in GDM samples

**Figure 1:**
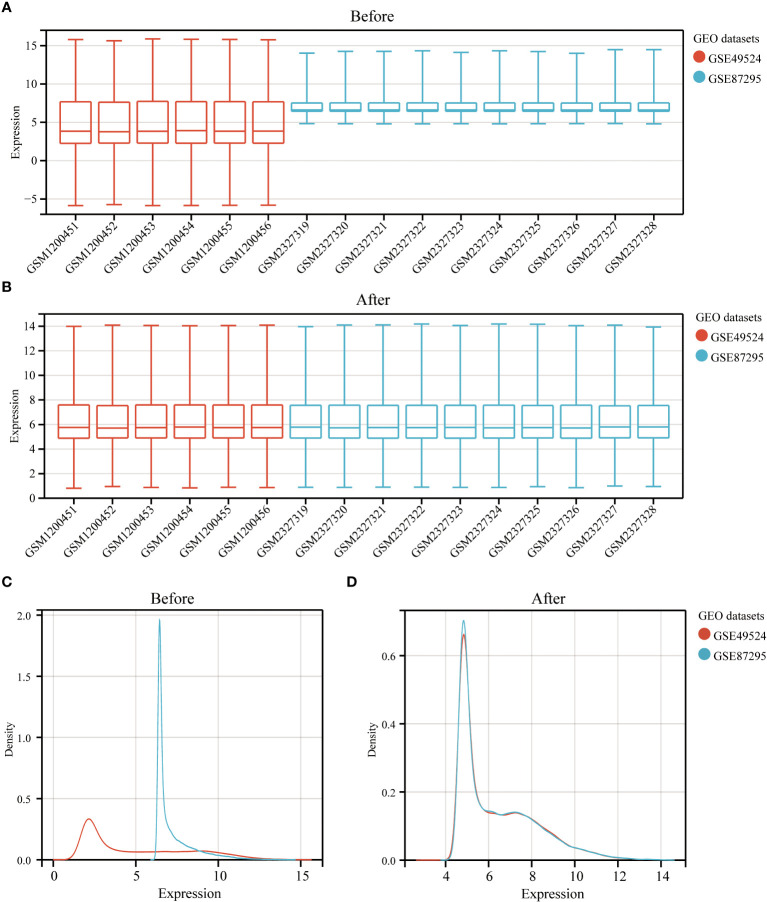
Normalization of GDM samples in selected GEO datasets. The box plots **(A, B)** and density curves **(C, D)** showed the gene expression distribution level in each GDM sample before and after removing the batch effect and normalization of samples.

**Figure 2:**
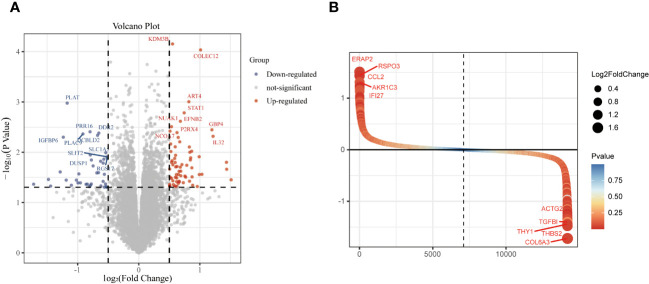
Identification of differentially expressed genes (DEGs) in GDM samples. The DEGs of GDM samples were shown in the volcano plot **(A)** and gene rank plot **(B)**.

The study design is presented in **Supplementary Figure 1**. The box plot shows that the expression profiles of GSE49524 and GSE87295 are normalized (**Figures 1A, B**). The density plot also revealed that the batch effect of the GSE49524 and GSE87295 meta-cohort was well removed (**Figures 1C, D**). Subsequently, according to the criteria (|log2 Fold change [FC]| >0.5 and p < 0.05), a total of 107 genes (68 upregulated and 39 down-regulated genes) were identified as the DEGs (**Table 2**), as shown in **Figures 2A, B**.

**Table 2:** The summary of differentially expressed genes (DEGs) in GDM.

Regulation	Count	Gene symbol
Up-regulated	68	MYO1D LAPTM5 LAP3 LIMCH1 NCOA7 PTPRB GCA GMPR PCSK7 NUAK1 TNFRSF14 SNCAIP KDM3B GFOD1 KCTD12 SLC40A1 SOX7 MYH10 FOXC1 ABLIM1 KIAA1324L TLE2 BMP4 FLI1 FAM107B HHEX PDGFB CHST15 P2RX4 MEOX2 TMC6 ATOH8 EMCN PLSCR4 EFNA1 TSPAN7 GBP2 SPINT2 FILIP1L EFNB2 C8orf4 PPP1R16B NTN4 RHOJ STAT1 SNCA PTGIS CPE ART4 PALMD EFEMP1 ALDH1A1 TNFSF10 GJA5 PTGS1 LMO2 CCDC58 SDPR BMP6 PLA2G4C PRKAR1A COLEC12 THBS1 GBP4 IL32 CCL2 RSPO3 ERAP2
Down-regulated	39	COL6A3 THBS2 THY1 KRT19 IGFBP6 PDGFRB PLAT CRISPLD2 TBX2 QPRT SLC2A3 CD248 PLAC9 SPON2 PITX1 KRT8 PRR16 DUSP1 CHPF ASNS OAF NCOR2 OBFC1 DCBLD2 MT1X DDR2 MME SMYD3 TNFRSF12A PLAUR HIPK2 DNAJB9 TUBB2B KRT34 SLC1A5 COL7A1 TBC1D2 SLIT2 RGS17

### Functional enrichment analysis for DEGs

**Figure 3:**
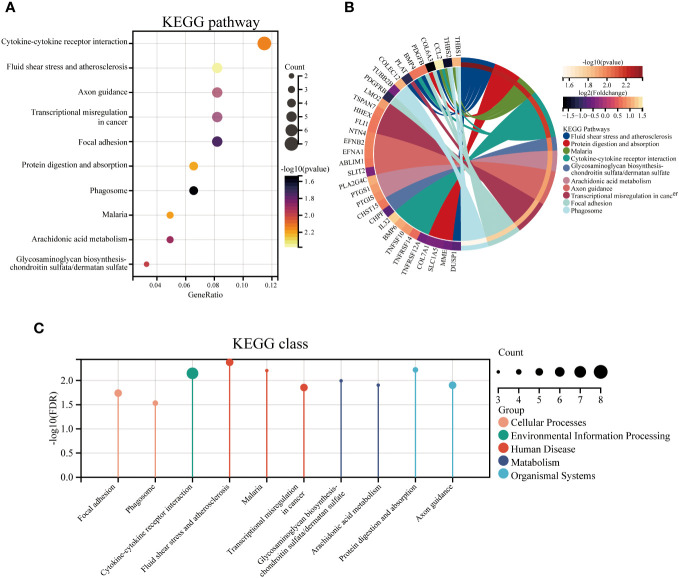
The top 10 enriched KEGG pathways for DEGs. The KEGG enrichment buddle diagram **(A)**, gene-pathway chord plot **(B)** and KEGG class lollipop plot **(C)**.

**Figure 4:**
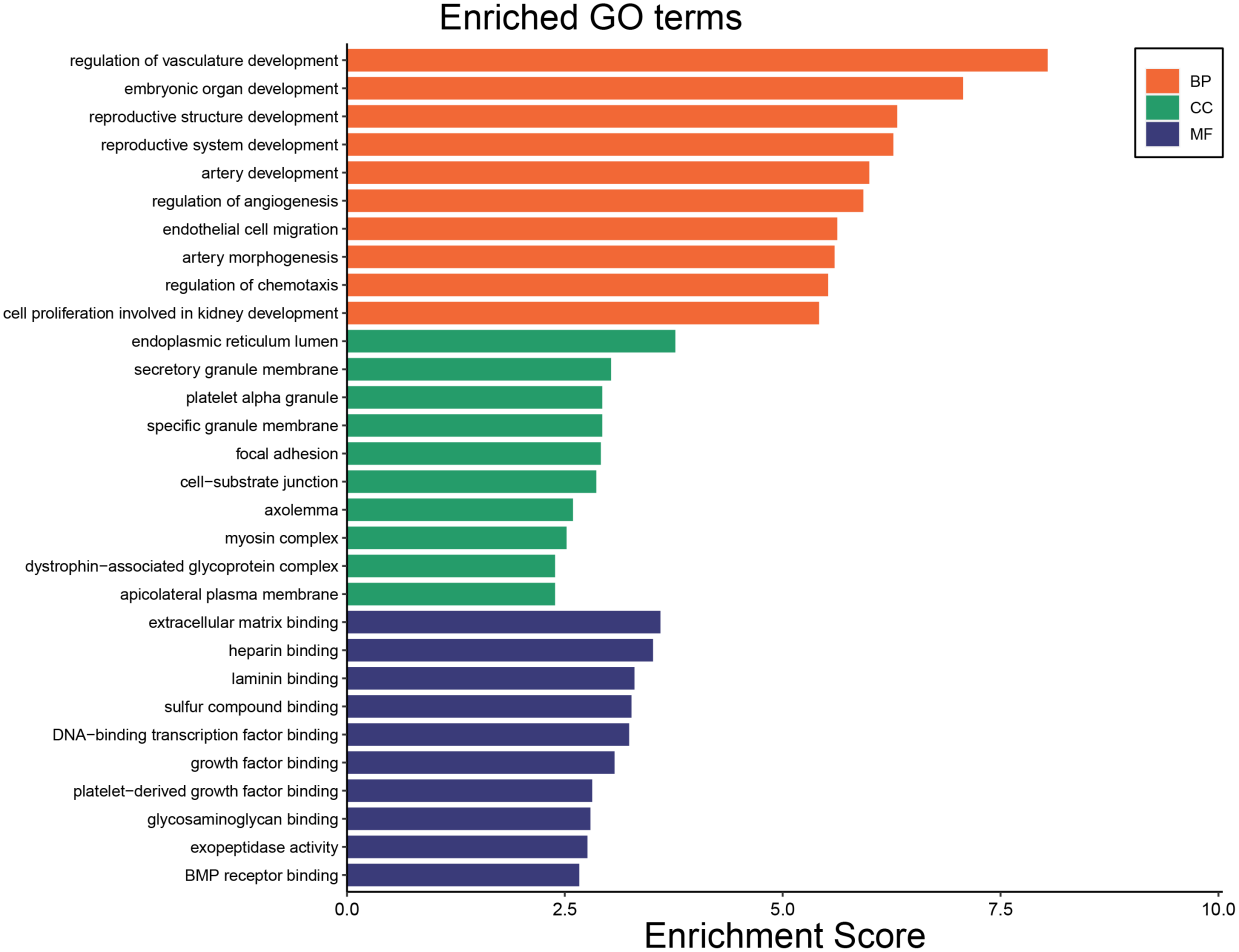
The top 10 enriched GO terms including GO BP, GO CC and GO MF.

**Figure 5:**
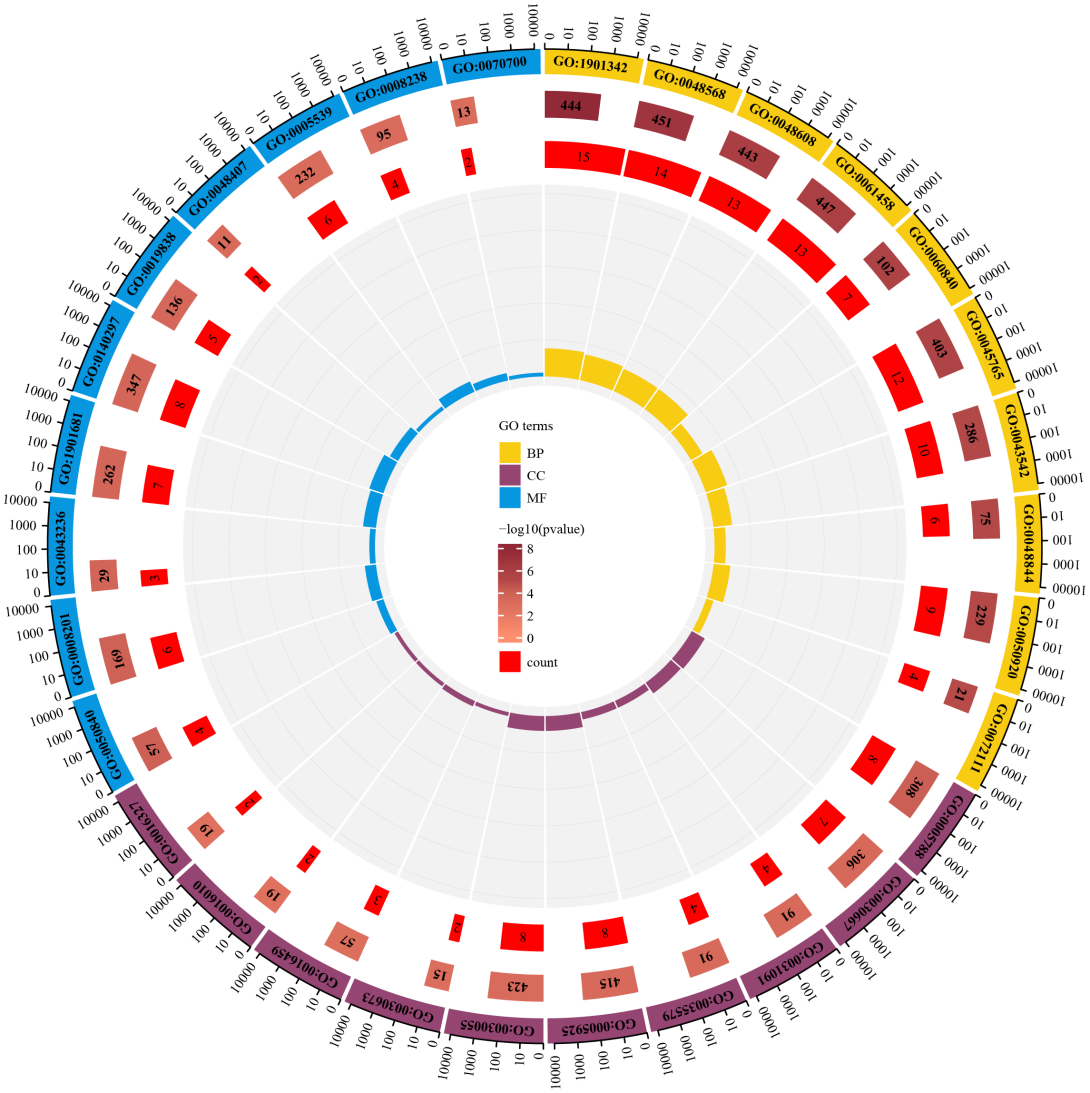
The enrichment circle map for enriched GO terms.

To identify the underlying molecular mechanism in GDM, the R package “clusterProfiler” was used to perform functional enrichment analysis, including KEGG pathway enrichment analysis (**Table 3**), three categories of GO functional enrichment analyses (BP, CC, MF) (**Table 4**) and DO enrichment analysis (**Table 5**), to further analyze the function of 107 DEGs. As illustrated in **Figure 3A**, the KEGG pathway analysis indicated that cytokine-cytokine receptor interaction, fluid shear stress and atherosclerosis, axon guidance, transcriptional misregulation in cancer and focal adhesion were the primarily enriched pathways in the DEGs (**Figure 3A**). The top 10 enriched KEGG pathway terms associations via ribbons to the participating DEGs genes are shown in a chord plot (**Figure 3B**). Then, these top 10 enriched KEGG pathways were mapped to their KEGG classes. As illustrated in **Figure 3C**, the KEGG enrichment bar plot showed that the DEGs covered the KEGG main class, involving cellular processes, environmental information processing, human disease, metabolism and organismal systems. The enriched GO terms for DEGs primarily included regulation of vasculature, embryonic organ, reproductive structure and reproductive system developments in the BP category; endoplasmic reticulum lumen, secretory granule membrane, platelet alpha granule and specific granule membrane in the CC category; and extracellular matrix, heparin, laminin and sulfur compound binding in the MM category (**Figures 4**, **5**).

**Table 3:** The results of enriched KEGG pathways.

ID	Description	pvalue	Gene symbol	Count
hsa05418	Fluid shear stress and atherosclerosis	0.0046542	PLAT/DUSP1/BMP4/PDGFB/CCL2	5
hsa05144	Malaria	0.0070435	THBS2/THBS1/CCL2	3
hsa04060	Cytokine-cytokine receptor interaction	0.0082339	TNFRSF12A/TNFRSF14/BMP4/TNFSF10/BMP6/IL32/CCL2	7
hsa04974	Protein digestion and absorption	0.0086849	COL6A3/MME/SLC1A5/COL7A1	4
hsa00532	Glycosaminoglycan biosynthesis - chondroitin sulfate/dermatan sulfate	0.0106172	CHPF/CHST15	2
hsa00590	Arachidonic acid metabolism	0.0121650	PTGIS/PTGS1/PLA2G4C	3
hsa04360	Axon guidance	0.0140842	SLIT2/ABLIM1/EFNA1/EFNB2/NTN4	5
hsa05202	Transcriptional misregulation in cancer	0.0177423	PLAT/FLI1/HHEX/TSPAN7/LMO2	5
hsa04510	Focal adhesion	0.0207677	COL6A3/THBS2/PDGFRB/PDGFB/THBS1	5
hsa04145	Phagosome	0.0315858	THBS2/TUBB2B/COLEC12/THBS1	4

**Table 4:** The results of enriched GO terms.

GO term	category	Description	pvalue	Count
GO:1901342	BP	regulation of vasculature development	9.12E-09	15
GO:0048568	BP	embryonic organ development	8.51E-08	14
GO:0048608	BP	reproductive structure development	4.86E-07	13
GO:0061458	BP	reproductive system development	5.38E-07	13
GO:0060840	BP	artery development	1.01E-06	7
GO:0045765	BP	regulation of angiogenesis	1.19E-06	12
GO:0043542	BP	endothelial cell migration	2.37E-06	10
GO:0048844	BP	artery morphogenesis	2.54E-06	6
GO:0050920	BP	regulation of chemotaxis	3.01E-06	9
GO:0072111	BP	cell proliferation involved in kidney development	3.83E-06	4
GO:0005788	CC	endoplasmic reticulum lumen	0.000171	8
GO:0030667	CC	secretory granule membrane	0.000934	7
GO:0031091	CC	platelet alpha granule	0.001178	4
GO:0035579	CC	specific granule membrane	0.001178	4
GO:0005925	CC	focal adhesion	0.001223	8
GO:0030055	CC	cell-substrate junction	0.00138	8
GO:0030673	CC	axolemma	0.002551	2
GO:0016459	CC	myosin complex	0.003019	3
GO:0016010	CC	dystrophin-associated glycoprotein complex	0.0041	2
GO:0016327	CC	apicolateral plasma membrane	0.0041	2
GO:0050840	MF	extracellular matrix binding	0.000253	4
GO:0008201	MF	heparin binding	0.000308	6
GO:0043236	MF	laminin binding	0.000503	3
GO:1901681	MF	sulfur compound binding	0.000544	7
GO:0140297	MF	DNA-binding transcription factor binding	0.000579	8
GO:0019838	MF	growth factor binding	0.00085	5
GO:0048407	MF	platelet-derived growth factor binding	0.001535	2
GO:0005539	MF	glycosaminoglycan binding	0.001612	6
GO:0008238	MF	exopeptidase activity	0.001742	4
GO:0070700	MF	BMP receptor binding	0.002162	2

**Table 5:** The results of enriched DO.

DO ID	Description	pvalue	geneID	Count
DOID:1115	sarcoma	3.2878E-05	TNFSF10/THBS2/THBS1/PLAUR/PDGFRB /PDGFB/FLI1/EFNB2/ALDH1A1	9
DOID:900	hepatopulmonary syndrome	0.00021204	CCL2/PLAT	2
DOID:6658	pulmonary large cell neuroendocrine carcinoma	0.00042176	KRT19/KRT8	2
DOID:9584	Venezuelan equine encephalitis	0.00042176	IL32/STAT1	2
DOID:11256	typhus	0.00069907	IL32/CCL2	2
DOID:13371	scrub typhus	0.00069907	IL32/CCL2	2
DOID:13476	supraglottis cancer	0.00069907	THBS2/KRT19	2
DOID:7763	carcinoma of supraglottis	0.00069907	THBS2/KRT19	2
DOID:1575	rheumatic disease	0.00101736	THBS1/SNCA/CCL2/PLAUR/PDGFRB /PDGFB/FLI1	7
DOID:418	systemic scleroderma	0.00101736	THBS1/SNCA/CCL2/PLAUR/PDGFRB /PDGFB/FLI1	7

**Figure 6:**
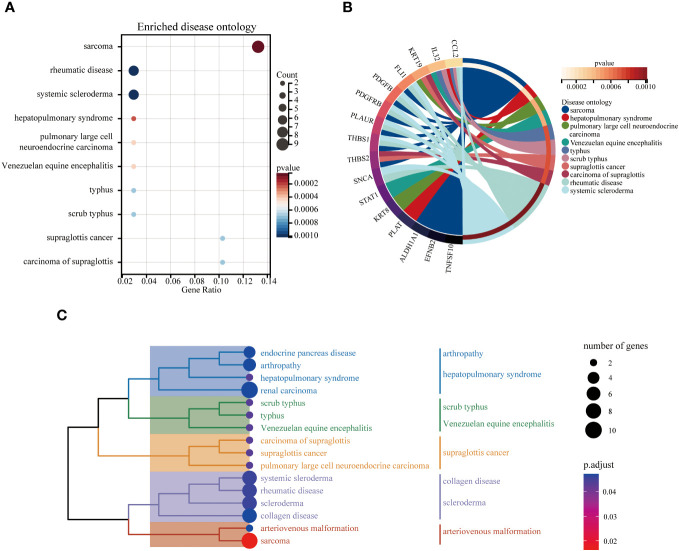
The top 10 enriched DO. The bubble plot showed the top 10 enriched DO for DEGs **(A)**. The chord plot showed the correlation between DEGs and DO **(B)**, and the tree plot showed the enriched DO classification **(C)**.

The DO analysis of DEGs depicted that sarcoma, rheumatic disease and systemic scleroderma are mainly enriched (**Figure 6A**). The top 10 enriched DO pathway terms associations via ribbons to the participating DEGs genes were shown in a chord plot (**Figure 6B**). The tree plot was used to classify enriched DO. As shown in **Figure 6C**, the DO enrichment tree plot demonstrated that the DEGs covered the DO main class, involving arthropathy, hepatopulmonary syndrome, scrub typhus, Venezuelan equine encephalitis, supraglottis cancer, collagen disease, scleroderma and arteriovenous malformation.

### The PPI network analysis

**Figure 7:**
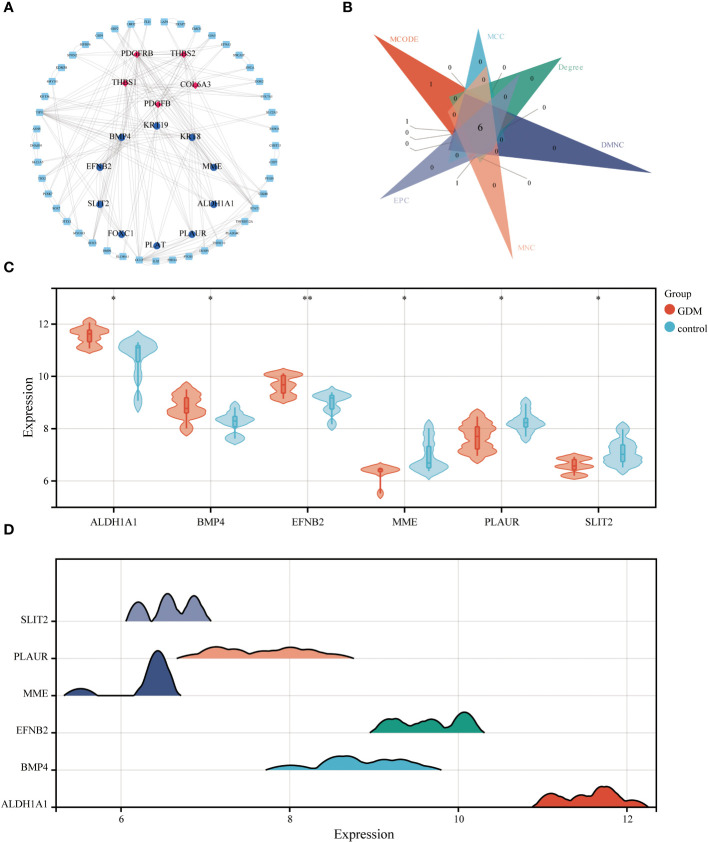
The PPI network analysis. The PPI network of DEGs, the genes with red (Cluster 1) and blue (Cluster 2) identified through MCODE algorithms **(A)**. Six algorithms were utilized to identify hub genes for GDM **(B)**. The differential expression level of 6 identified hub genes in GDM and control samples **(C)**. The expression distribution of 6 identified hub genes in GDM **(D)** (*p<0.05, **p<0.01).

The STRING database and Cytoscape software were utilized to construct the PPI network of DEGs in GDM, as illustrated in **Figure 7A**. The six common hub DEGs (including *ALDH1A1*, *BMP4*, *EFNB2*, *MME*, *PLAUR* and *SLIT2*) were ascertained using six algorithms (including MCODE, MCC, Degree, DMNC, MNC and EPC) in cytoHubba, as shown in **Figure 7B**, **Table 6**. Subsequently, the relative expressions of these six common hub DEGs were assessed and compared in the GDM and normal samples. Analysis of expression profiles indicated that *ALDH1A1*, *BMP4* and *EFNB2* were significantly higher in the patients with GDM compared with the normal samples (P<0.05 for *ALDH1A1* and *BMP4*; P<0.01 for *EFNB2*) and *MME*, *PLAUR* and *SLIT2* expression in normal samples was higher than that in patients with GDM (P<0.05 for *MME*, *PLAUR* and *SLIT2*) (**Figure 7C**). The function of these six hub genes and expression distribution of six identified hub genes in GDM were further investigated, and results showed the expression degree of these six hub genes s in patients with GDM in the following order as *ALDH1A1* > *EFNB2* > *BMP4* > *PLAUR* > *SLIT2* > *MME* (**Figure 7D**).

**Table 6:** The information of identified hub genes.

Number	Gene symbol	Description	Regulation
1	ALDH1A1	aldehyde dehydrogenase 1 family member A1	Up-regulated
2	BMP4	bone morphogenetic protein 4	Up-regulated
3	EFNB2	ephrin B2	Up-regulated
4	MME	membrane metalloendopeptidase	Down-regulated
5	PLAUR	plasminogen activator, urokinase receptor	Down-regulated
6	SLIT2	slit guidance ligand 2	Down-regulated

### Correlation between each of the hub genes

**Figure 8:**
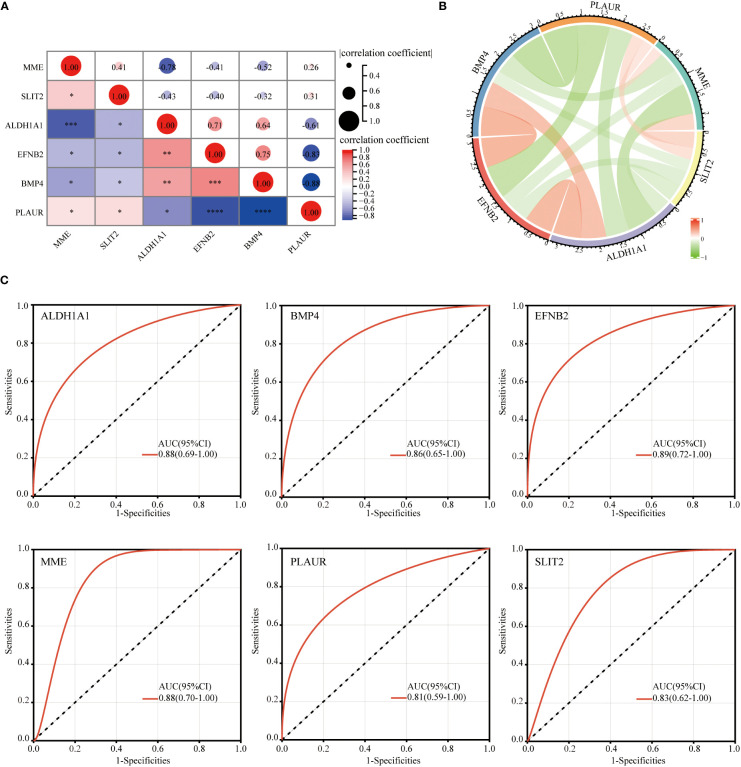
Correlation between each of hub genes and ROC curve analysis. The correlation heatmap **(A)** and circle plot **(B)** of 6 identified hub genes. ROC curve diagnostic analysis for evaluating 6 identified hub genes as GDM biomarkers **(C)** (*p<0.05, **p<0.01, ***p<0.005, ****p<0.001).

After identifying these six hub DEGs, the relationship between the expression levels of hub genes was analyzed and depicted using Spearman’s correlation analysis. As shown in **Figures 8A, B**, both the correlation heatmap and circle plot indicated that six hub genes were all significantly correlated with each other.

### ROC curve analysis

The ROC analysis was performed to evaluate the potential of these six hub DEGs as biomarkers for GDM. ROC curve analysis confirmed that AUC was 0.88 (95% confidence interval [CI], 0.69–1.00) for *ALDH1A1*, 0.86 (95% CI, 0.65–1.00) for *BMP4*, 0.89 (95% CI, 0.72–1.00) for *EFNB2*, 0.88 (95% CI, 0.70–1.00) for *MME*, 0.81 (95% CI, 0.59–1.00) for *PLAUR* and 0.83 (95% CI, 0.62–1.00) for *SLIT2* (**Figure 8C**). AUC value >0.8 was considered to be statistically significant, which confirmed the accuracy of our risk model.

### Immune infiltration analysis of the six hub DEGs in GDM

**Figure 9:**
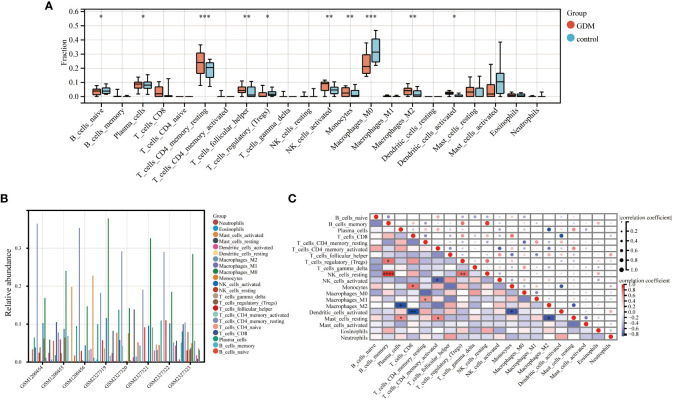
Immune infiltration analysis. The immune infiltration level of 22 immune cells in GDM and control samples **(A)**. The relative abundance of 22 immune cells in GDM samples **(B)**. The correlation between each of 22 immune cells in GDM samples **(C)**. (*p<0.05, **p<0.01, ***p<0.005, ****p<0.001)..

The CIBERSORT algorithm by R package “IOBR” was performed to accomplish the quantitative immune infiltration analysis of 22 immune cells in GDM and normal samples to further explore the differences in immune cell infiltration in GDM samples and normal tissues. The result demonstrated that the GDM group had higher infiltration of plasma cells, resting CD4^+^ memory cells, T follicular helper cells, activated NK cells, monocytes, M2 macrophage cells and activated dendritic cells than the normal group, while the normal group had higher naive B cells, M0 macrophage cells and regulatory T cells (Tregs) (**Figure 9A**). The relative abundance of the 22 kinds of immune cells in the GDM was also analyzed (**Figure 9B**). In addition, Spearman’s correlation analysis of immune cell abundance revealed the relationship between immune cells (**Figure 9C**). From the correlation matrix, the positive correlation between B cells memory and NK cells resting was the strongest, while the obvious negative correlation existed between plasma cells and macrophage M2, between T cell CD8 and dendritic cells activated (**Figure 9C**).

### The correlation between the hub genes and immune cells

**Figure 10:**
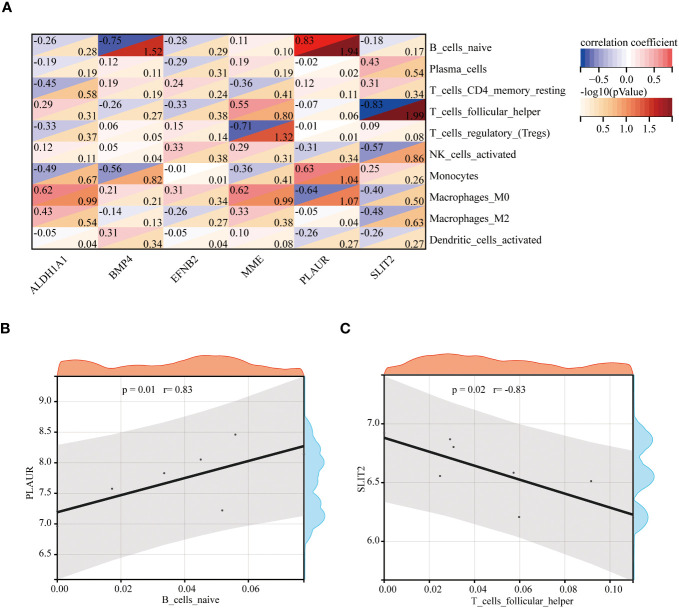
The correlation between the hub genes and the immune cells. The correlation heatmap of 6 hub genes and 10 differentially infiltrating immune cells **(A)**. The strongest positive **(B)** and negative **(C)** correlation between hub gene and immune cell.

Spearman’s correlation analysis was used to depict the correlation between the immune cells and these six hub genes to further investigate immune microenvironment-related potential biomarkers for patients with GDM (**Figure 10A**). Based on the results of correlation analysis, *PLAUR* displayed the strongest positive correlation with B cells naive (r = 0.83, p = 0.01) (**Figure 10B**) and strongest negative correlation with T cells follicular helper (r = −0.83, p = 0.02) (**Figure 10C**).

## Discussion

GDM is a critical obstetric complication of pregnancy caused by both genetic and environmental factors (21). Maternal exposure to hyperglycemia leads to glucose stress response and concurrent systemic low-grade inflammation, which involves altered infiltration, differentiation and activation of maternal innate and adaptive immune cells (22). Many studies demonstrated that immune dysfunction induced by hyperglycemia plays a vital role in the development of GDM (23). However, the significance of immune dysfunction and immune-related hub genes in the GDM pathophysiology remains ambiguous. Thus, identifying novel immune-related molecular mechanisms and effective molecular targeted therapies is essential in determining effective GDM treatment strategies along with GDM prevention programs.

In the long term, sustained hyperglycemia can trigger glucose homeostasis, chronic dysregulation (24) and maternal immune imbalance owing to chronic hyperglycemia. This leads to the deterioration of the disease and the further reduction of maternal insulin sensitivity (22). From this point of view, the human primary endothelial cells (HUVECs) collected from the umbilical cord of GDM mothers are an expedient measure to study the hub immune-related biomarkers and their impact on immunity in patients with GDM. In our study, the evidence from the analysis of differential expression profiles of HUVECs from umbilical cords of GDM demonstrated that 107 DEGs were identified between GDM mothers and the mother without GDM. The KEGG analysis depicted that these DEGs were the most significantly enriched in cytokine-cytokine receptor interaction, which was similar to previous studies showing that the interaction pathway was elevated in the fecal microbiota of patients with GDM (25). Moreover, these DEGs were also enriched in fluid shear stress and atherosclerosis, axon guidance, transcriptional misregulation in cancer and focal adhesion in the KEGG pathway analysis. Zhu et al. demonstrated that axon guidance enriched the KEGG pathway in GDM arterial endothelial cell samples (26). The GO analysis revealed that its molecular function was related to the vasculature, embryonic organ, reproductive structure and reproductive system development, suggesting that sustained hyperglycemia in patients with GDM was related to embryonic development. We also performed the DO analysis to explore the function of 107 DEGs, and the results revealed these DEGs also were the most significantly enriched in sarcoma. Currently, no studies have shown a correlation between sarcoma and GDM. A case report indicated that an infant whose mother had diabetes had inborn cardiac sarcomas (27). Although there is a lack of evidence, it undoubtedly further suggests that maternal GDM may have harmful effects on embryonic development.

After identifying the function of 107 DEGs, the scope was narrowed down using six algorithms of cytoHubaa to further determine the hub DEGs and performed the ROC analysis to assess the accuracy of the hub DEGs as the diagnostic biomarkers in patients with GDM. The results showed that the AUC value of these six hub DEGs (*ALDH1A1*, *BMP4*, *EFNB2*, *MME*, *PLAUR* and *SLIT2*) was > 0.8, which suggested these six hub DEGs can serve as diagnostic biomarkers for distinguishing patients with GDM from normal individuals. To further screen hub genes related to the immune microenvironment, we evaluated the association between the expression of hub genes and immune cell infiltration. According to the analysis, *PLAUR* was the most significant and strongest positive correlation with naive B cells, and *SLIT2* was the most significant and strongest negative with T follicular helper cells. In this study, *PLAUR* and *SLIT2* were considered as the two potential immune-related biomarkers. Compared to the umbilical cords sample from the mother without GDM, *PLAUR* and *SLIT2* were down-regulated in the GDM samples, and naive B cell, as well as T cells follicular helper, were upregulated, which suggested *PLAUR* may be a promoting factor for B cell naive, while *SLIT2* may be a negative factor for T cells follicular helper in GDM samples.

The PLAUR participates in regulating various physiological and pathological processes, including cellular adhesion, cell motility and angiogenesis (28). Few reports showed the relationship between *PLAUR* and GDM. Our study first proposed the relationship between *PLAUR* and immune regulation in GDM. SLIT2 is a regulator of inflammatory response and glucose metabolism. Kang et al. indicated that circulating SLIT2 level was negatively correlated with serum glucose in patients with diabetes (29). Kang et al. reported that SLIT2 level in the maternal peripheral blood in patients with GDM was negatively associated with blood glucose in neonates (30). Our study also proposed the relationship between *SLIT2* and immune regulation in GDM.

In conclusion, we comprehensively analyzed the gene expression profile based on the RNA-seq data with umbilical cords of eight GDM and eight mothers without GDM from the GEO database and evaluated the function, diagnostic value and immune infiltration of hub DEGs in GDM. Our analysis pointed out six hub DEGs with high diagnostic value, and among them, *PLAUR* and *SLIT2* were considered as two biomarkers, which had the strongest correlation with B cells naïve and T cells follicular helper, respectively. However, these results should be further validated in animal models with GDM in the future, and further investigation into the molecular functions of immune-related hub genes may facilitate a better understanding of more efficient treatment strategies against GDM.

## Data availability statement

The datasets presented in this study can be found in online repositories. The names of the repository/repositories and accession number(s) can be found in the article/**Supplementary Material**.

## Author contributions

JC: Data curation, Formal analysis, Writing – original draft. HD: Data curation, Formal analysis, Writing – original draft. XK: Formal analysis, Writing – original draft. HC: Writing –original draft, Writing – review & editing. JW: Writing – review & editing, Writing – original draft.

## Supplementary material

Supplementary Figure 1 The study design.
