# Supplementary figures and images for: The integrated bioinformatic analysis identifies immune microenvironment-related potential biomarkers for patients with gestational diabetes mellitus
Source: Front Immunol. 2024 Feb 21;15:1296855. doi: 10.3389/fimmu.2024.1296855 (PMC10917066; doi:10.3389/fimmu.2024.1296855)

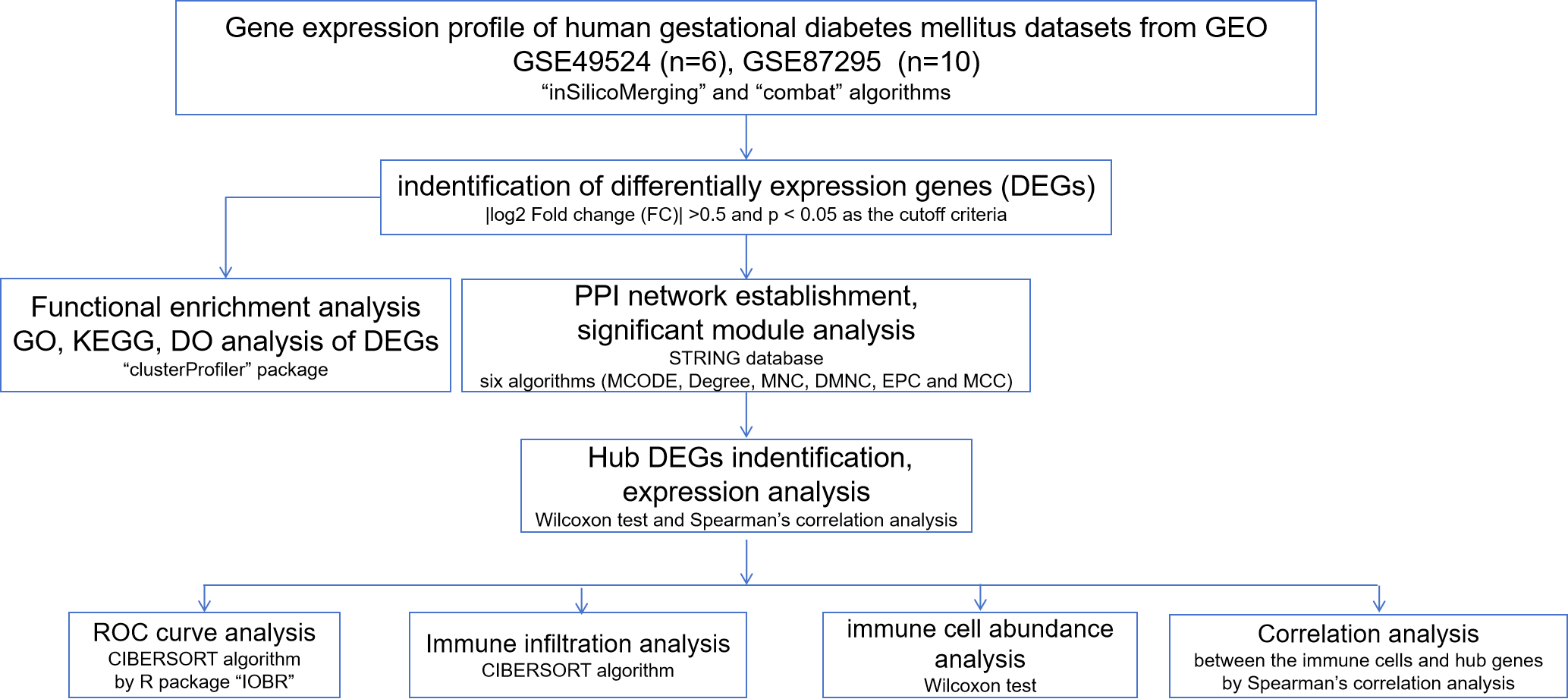

Supplement: Supplementary Figure 1 — The study design. [file Image_1.tif]
